# Supplementary material for: A Methodological Approach to Use Contextual Factors for Epidemiological Studies on Chronic Exposure to Air Pollution and COVID-19 in Italy
Source: Int J Environ Res Public Health. 2022 Mar 1;19(5):2859. doi: 10.3390/ijerph19052859 (PMC8910469; doi:10.3390/ijerph19052859)
Supplement: Supplementary file 1 [file ijerph-19-02859-s001.zip › ijerph-1597956-supplementary.pdf]

Table S1. Contextual variables at municipality level in Italy: name, label, data source, temporal dimension and description (7903 municipalities)

General features

Variable	Label	Source/link	Year	Description
Municipality code	code	Italian National Institute of Statistics, ISTAT	2020	ISTAT code of the Municipality
Municipality name	mun	Italian National Institute of Statistics, ISTAT	2020	Name of the municipality
Province code	cod_pro	Italian National Institute of Statistics, ISTAT	2020	ISTAT code of the Province
Province name	den_pcm	Italian National Institute of Statistics, ISTAT	2020	Name of the Province
Region code	cod_reg	Italian National Institute of Statistics, ISTAT	2020	ISTAT code of the Region
Region name	region	Italian National Institute of Statistics, ISTAT	2020	Name of the Region
Automobile abbreviation	aut_abbr	Italian National Institute of Statistics, ISTAT	2020	Automobile abbreviation of the Region
Po valley	po_valley	Italian National Institute of Statistics, ISTAT	2020	Dichotomous variable that indicates whether the regions and their municipalities belongs to the Po valley or not (Piedmont, Lombardy, Trentino-Alto Adige, Veneto, Friuli-Venezia Giulia, Emilia-Romagna)
Geographical areas	geo_areas	Italian National Institute of Statistics, ISTAT	2020	North (Piedmont, Valle d'Aosta, Lombardy, Trentino-Alto Adige, Veneto, Friuli - Venezia Giulia, Liguria, Emilia-Romagna), Center (Tuscany,; Marche, Umbria, Lazio) and South (Abruzzo, Molise, Campania, Puglia, Basilicata, Calabria, Sicily, Sardinia).

First dimension: geographic characteristics

Variable	Label	Source/link	Year	Description
Area	area	Italian National Institute of Statistics, ISTAT (https://www.istat.it/it/archivio/156224)	2020	Size (km2)

Elevation	elevation	Italian National Institute of Statistics, ISTAT (https://www.istat.it/it/archivio/156224)	2020	Elevation (m)
Altimetric zone	zon_altimetric	Italian National Institute of Statistics, ISTAT (https://www.istat.it/it/archivio/156224)	2020	1 = Inland mountains; 2 = Coastal mountains; 3 = Inland hills; 4 = Coastal hills; 5 = Plane
Coastal	coastal	Italian National Institute of Statistics, ISTAT (https://www.istat.it/it/archivio/156224)	2020	Municipality on the coast (yes/no)
Island	island	Italian National Institute of Statistics, ISTAT (https://www.istat.it/it/archivio/156224)	2020	Municipality on an island (yes/no)
Urbanization	urbanizzaztio	EUROSTAT (https://www.istat.it/it/archivio/156224)	2018	1 = Major city; 2 = Minor city; 3 = Sub-urban or rural area
Coastal area	coastal_area	EUROSTAT (https://www.istat.it/it/archivio/156224)	2018	1 = Coastal areas, municipalities located on the coast or having at least 50% of the surface at a distance from the sea of less than 10 km; 0 = Non-coastal areas
Longitude	mun_lon	Italian National Institute of Statistics, ISTAT	2011	Longitude
Latitude	mun_lat	Italian National Institute of Statistics, ISTAT	2011	Latitude

Second dimension: demographic and anthropogenic characteristics

Population 2019	population_2019	Italian National Institute of Statistics, ISTAT (https://www.istat.it/it/archivio/156224)	2011	Resident population, Oct 9 2011 (Census date)
Population 2011	population_2011	Italian National Institute of Statistics, ISTAT (https://www.istat.it/it/archivio/156224)	2019	Resident population, Dec 31 2019
Population class	pop_class	Italian National Institute of Statistics, ISTAT (https://www.istat.it/it/archivio/156224)	2020	Population size, in classes: 1=<2.000; 2=2.000-10.000; 3=10.000-50.000, 4=>50.000
Pop density	pop_density	Italian National Institute of Statistics, ISTAT (https://www.istat.it/it/archivio/156224)	2019	Population density (pop.2019 / size of the area)
% over 65 years	over65	ISTAT (http://demo.istat.it/popres/download.php?anno=2020&lingua=ita)	2020	% pop over 65 years
Population maximum	pop_maximun	Italian National Institute of Statistics, ISTAT	2011	Population density (maximum in the cell 1 km2)
Impervious Surface Areas	ISA	COPERNICUS	2012	Impervious Surface Areas (maximum in the cell 1 km2)
Light at Night	LAN	NASA	2012	Light at Night (maximum in the cell 1 km2)
Percentage of urban coverage	pcturb	CORINE (European Enviromental Agency)	2012	Percentage of urban coverage (maximum in the cell 1 km2)

Length of the roads	l_roads	TeleAtlas TomTom	2012	Length of the roads (maximum in the cell 1 km2)

Third dimension: mobility

Variable	Label	Source/link	Year	Description
Attraction index	attraction_index	ISTAT - AR.CHI.M.E.DE (http://amisuradicomune.istat.it/aMisuraDiComune/)	mean 2014-2015	Ratio between movements of individuals who work or study in the municipality, and total individuals in the area
Self-containment index	self_cont_index	ISTAT - AR.CHI.M.E.DE (http://amisuradicomune.istat.it/aMisuraDiComune/)	mean 2014-2015	Ratio between individuals who work or study in the municipality, and total movements in the area
Movements in 2019	movements_2019	ASSAEROPORTI (https://assaeroporti.com/dati-annuali/)	2019	Number of flights, average 2019
Passengers in 2019	passengers_2019	ASSAEROPORTI (https://assaeroporti.com/dati-annuali/)	2019	Number of passengers, average 2019
Movements in 2020	movements_gen2020	ASSAEROPORTI (https://assaeroporti.com/dati-annuali/)	january 2020	Number of flights at January 2020
Passengers in 2020	passengers_gen2020	ASSAEROPORTI (https://assaeroporti.com/dati-annuali/)	january 2020	Number of passengers at January 2020
Movements out municipality	mov_extra	Italian National Institute of Statistics, ISTAT (https://www.istat.it/it/archivio/139381)	2011	Number of individuals who move outboud the municipality for work or study reasons
Movements in municipality	mov_intra	Italian National Institute of Statistics, ISTAT (https://www.istat.it/it/archivio/139381)	2011	Number of individuals who move inside the municipality for work or study reasons
Total movements	mov_tot	Italian National Institute of Statistics, ISTAT (https://www.istat.it/it/archivio/139381)	2011	Number of individuals who move for work or study reasons
Local Work Systwem index	SLL	Italian National Institute of Statistics, ISTAT (https://www.istat.it/it/archivio/252261)	2020	Code of Local Work Systwem (Sistema Locale del Lavoro, SLL)
train_stations	train_stations	OpenStreetMap.org (http://www.datiopen.it/it/opendata/Mappa_delle_stazioni_ferroviarie_in_Italia)	2016	Presence of a rail station (yes/no)
Railway stations	n_railway_station	OpenStreetMap.org (http://www.datiopen.it/it/opendata/Mappa_delle_stazioni_ferroviarie_in_Italia)	2016	Number of rail stations
Airports within 30 km from the municipality	airports_30km	Italian National Institute of Statistics, ISTAT (https://www4.istat.it/it/archivio/41899)	2015	Number of airports within 30-km buffer

Fourth dimension: socio-economic and health characteristics

Variable	Label	Source/link	Year	Description
Income	income	ISTAT - ARCH.I.M.E.DE (http://amisuradicomune.istat.it/aMisuraDiComune/)	Mean 2014-2015	Ratio between gross income and number of households components (euro)
Entrepreneurship rate	entrepr_rate	ISTAT - ASIAImprese (http://amisuradicomune.istat.it/aMisuraDiComune/)	mean 2014-2015	Number of enterprises, per 1000 inhabitants
Socio-Economic Deprivation index - continuous variable	SEP	Italian Institute of Health (ISS)	2011	Socio-Economic Deprivation index, continuous
Socio-Economic Deprivation index	SEP_cat	Italian Institute of Health (ISS)	2011	Socio-Economic Deprivation index, in quintiles
Hospitalization rate for circulatory causes	rate_R_cir	Statistics Service of ISS, based on official data of Ministry of Health, in compliance with the Regulation (EU) 2016/679 for General Data Protection (GDPR)	annual average 2013-2018	Hospitalization rate for circulatory causes (annual average per 100)
Hospitalization rate for respiratory causes	rate_R_res	Statistics Service of ISS, based on official data of Ministry of Health, in compliance with the Regulation (EU) 2016/679 for General Data Protection (GDPR)	annual average 2013-2018	Hospitalization rate for respiratory causes (annual average per 100)
Hospitalization rate for all causes	rate-R_tot	Statistics Service of ISS, based on official data of Ministry of Health, in compliance with the Regulation (EU) 2016/679 for General Data Protection (GDPR)	annual average 2013-2018	Hospitalization rate for all causes (annual average per 100)
Mortality rate for circulatory causes	rate_M_cir	Statistics Service of ISS, based on official data of ISTAT, in compliance with the Regulation (EU) 2016/679 for General Data Protection (GDPR)	annual average 2013-2017	Mortality rate for circulatory causes (annual average per 100)
Mortality rate for respiratory causes	rate_M_res	Statistics Service of ISS, based on official data of ISTAT, in compliance with the Regulation (EU) 2016/679 for General Data Protection (GDPR)	annual average 2013-2017	Mortality rate for respiratory causes (annual average per 100)
Mortality rate for all causes	rate_M_tot	Statistics Service of ISS, based on official data of ISTAT, in compliance with the Regulation (EU) 2016/679 for General Data Protection (GDPR)	annual average 2013-2017	Mortality rate for all causes (annual average per 100)

Fifth dimension: availability of health care

Variable	Label	Source/link	Year	Description
Teaching hospitals	num_polyclinics	Italian Ministry of Health (http://www.dati.salute.gov.it/dati/dettaglioDataset.jsp?menu=dati&idPag=96)	2019	Numbers of Polyclinics in the municipality

General hospitals	num_hosp	Italian Ministry of Health (http://www.dati.salute.gov.it/dati/dettaglioDataset.jsp?menu=dati&idPag=96)	2019	Numbers of Hospital in the municipality
Accredited private nursing homes	num_ircss	Italian Ministry of Health (http://www.dati.salute.gov.it/dati/dettaglioDataset.jsp?menu=dati&idPag=96)	2019	Numbers of IRCSS and public and private foundations in the municipality
Nursing homes	num_nh	Italian Ministry of Health (http://www.dati.salute.gov.it/dati/dettaglioDataset.jsp?menu=dati&idPag=96)	2019	Numbers of Nursing homes in the municipality
Number of acute care beds	nbeds_acute_ord	Italian Ministry of Health (http://www.dati.salute.gov.it/dati/dettaglioDataset.jsp?menu=dati&idPag=96)	2019	Number of acute beds in ordinary regimen wards in the municipality
Number of acute beds to pay	nbeds_acute_pay	Italian Ministry of Health (http://www.dati.salute.gov.it/dati/dettaglioDataset.jsp?menu=dati&idPag=96)	2019	Number of acute beds to pay in the municipality
Number of acute beds in day hospital wards	nbeds_acute_dhos	Italian Ministry of Health (http://www.dati.salute.gov.it/dati/dettaglioDataset.jsp?menu=dati&idPag=96)	2019	Number of acute beds in day hospital regimen wards in the municipality
Number of acute beds in day surgery wards	nbeds_acute_dsurg	Italian Ministry of Health (http://www.dati.salute.gov.it/dati/dettaglioDataset.jsp?menu=dati&idPag=96)	2019	Number of acute beds in day surgery regimen wards in the municipality
Number of long-term hospital beds	nbeds_lstay_ord	Italian Ministry of Health (http://www.dati.salute.gov.it/dati/dettaglioDataset.jsp?menu=dati&idPag=96)	2019	Number of beds in ordinary long-stay wards in the municipality
Number of beds to pay in long-stay wards	nbeds_lstay_pay	Italian Ministry of Health (http://www.dati.salute.gov.it/dati/dettaglioDataset.jsp?menu=dati&idPag=96)	2019	Number of beds to pay in long-stay wards in the municipality
Number of beds in long-stay wards	nbeds_lstay_dhos	Italian Ministry of Health (http://www.dati.salute.gov.it/dati/dettaglioDataset.jsp?menu=dati&idPag=96)	2019	Number of beds in long-stay wards in the municipality
Number of beds in day surgery long-stay wards	nbeds_lstay_dsurg	Italian Ministry of Health (http://www.dati.salute.gov.it/dati/dettaglioDataset.jsp?menu=dati&idPag=96)	2019	Number of beds in day surgery long-stay wards in the municipality
Number of long-term hospital beds	nbeds_rehab_ord	Italian Ministry of Health (http://www.dati.salute.gov.it/dati/dettaglioDataset.jsp?menu=dati&idPag=96)	2019	Number of beds in ordinary rehabilitation wards in the municipality

Number of beds to pay in rehabilitation wards	nbeds_rehab_pay	Ministero della Salute (http://www.dati.salute.gov.it/dati/dettaglioDataset.jsp?menu=dati&idPag=96)	2019	Number of beds to pay in rehabilitation wards in the municipality
Number of beds in rehabilitation wards	nbeds_rehab_dhos	Italian Ministry of Health (http://www.dati.salute.gov.it/dati/dettaglioDataset.jsp?menu=dati&idPag=96)	2019	Number of beds in rehabilitation wards in the municipality
Number of beds in day surgery in rehabilitation wards	nbeds_rehab_dsurg	Italian Ministry of Health (http://www.dati.salute.gov.it/dati/dettaglioDataset.jsp?menu=dati&idPag=96)	2019	Number of beds in day surgery in rehabilitation wards in the municipality
Number of beds in ordinary Intensive Care Units	nbeds_ICU_ord	Italian Ministry of Health (http://www.dati.salute.gov.it/dati/dettaglioDataset.jsp?menu=dati&idPag=96)	2019	Number of beds in ordinary Intensive Care Units in the municipality
Number of beds to pay in Intensive Care Units	nbeds_ICU_pay	Italian Ministry of Health (http://www.dati.salute.gov.it/dati/dettaglioDataset.jsp?menu=dati&idPag=96)	2019	Number of beds to pay in Intensive Care Units in the municipality
Number of beds in rehabilitation Intensive Care Units	nbeds_ICU_dhos	Italian Ministry of Health (http://www.dati.salute.gov.it/dati/dettaglioDataset.jsp?menu=dati&idPag=96)	2019	Number of beds in rehabilitation Intensive Care Units in the municipality
Number of beds in day surgery in Intensive Care Units	nbeds_ICU_dsurg	Italian Ministry of Health (http://www.dati.salute.gov.it/dati/dettaglioDataset.jsp?menu=dati&idPag=96)	2019	Number of beds in day surgery in Intensive Care Units in the municipality
Number of Emergecny Departments	n_ps	Italian Ministry of Health (http://www.salute.gov.it/portale/documentazione/p6_2_8_1_1.jsp?lingua=italiano&id=17)	2019	Number of Emergecny Rooms in the municipality
Number of family counseling	n_fam_counseling	Italian Ministry of Health (http://www.dati.salute.gov.it/dati/dettaglioDataset.jsp?menu=dati&idPag=70)	2019	Number of family counseling in the municipality
Distance (m) from closest polyclinical	dist_ao	Elaboration on Italian Ministry of Health data	2019	Distance (m) from closest polyclinical
Distance (m) from closest hospital	dist_osp	Elaboration on Italian Ministry of Health data	2019	Distance (m) from closest hospital

Distance (m) from closest IRCSS or public or private foundations	dist_ircss	Elaboration on Italian Ministry of Health data	2019	Distance (m) from closest IRCSS or public or private foundations
Distance (m) from closest nursing home	dist_cc	Elaboration on Italian Ministry of Health data	2019	Distance (m) from closest nursing home
Distance (m) from closest health care facility	dist_healthcare_facility	Elaboration on Italian Ministry of Health data	2019	Distance (m) from closest health care facility
Distance (m) from closest Emergency Room	dist_er	Elaboration on Ministero della Salute data	2019	Distance (m) from closest Emergency Room
Number of nursing residences	n_healthcare_residences	Elaboration on ISTAT data (archive ASIA Unità Locali)	2017	Number of healthcare residences in the municipality
Number of workers in healthcare residences	workers_heacareres	Elaboration on ISTAT data (archive ASIA Unità Locali)	2017	Number workers in healthcare residences in the municipality
